# A Review on Sex Steroid Hormone Estrogen Receptors in Mammals and Fish

**DOI:** 10.1155/2020/5386193

**Published:** 2020-02-07

**Authors:** Eric Amenyogbe, Gang Chen, Zhongliang Wang, Xiaoying Lu, Mingde Lin, Ai Ying Lin

**Affiliations:** ^1^College of Fisheries, Guangdong Ocean University, Zhanjiang 524025, China; ^2^Guangdong Provincial Key Laboratory of Aquaculture in the South China Sea for Aquatic Economic Animal of Guangdong Higher Education Institutes, Laboratory of Fish Aquaculture, Zhanjiang 524025, China

## Abstract

Steroid hormones play essential roles in the reproductive biology of vertebrates. Estrogen exercises its effect through estrogen receptors and is not only a female reproductive hormone but acts virtually in all vertebrates, including fish, and is involved in the physiological and pathological states in all males and females. Estrogen has been implicated in mandible conservation and circulatory and central nervous systems as well as the reproductive system. This review intended to understand the structure, function, binding affinities, and activations of estrogens and estrogen receptors and to discuss the understanding of the role of sex steroid hormone estrogen receptors in mammals and fish.

## 1. Introduction

Estrogens are steroid hormones and are largely incorporated in ovary and testis [1]. Estrogens employ their effects mainly through cytosolic estrogen receptors (ERs) existing in target tissues [2]. Local production of steroid hormones in the brain and other tissues of importance is of increasing interest concerning control of numerous behavioral and physiological processes from sexual behavior to brain sex differentiation in a variety of species [3]. While mammals have one gene for double estrogen receptor subtypes (ESR1 and ESR2), in teleost fishes, three estrogen receptors of the regulatory transcription factor type have been acknowledged, a single ER*α* and two ER*β*1 and ER*β*2 genes [4, 5]. Estrogen is renowned for its involvement in the dictate of “oogenesis, vitellogenesis, gonadotropin regulation, testicular growth, and other facets of regeneration, in addition to having controlling roles in several structure organizations.” [6–10] In this review, we aim at understanding the structure, function, binding affinities, and activations of estrogens and estrogens receptors and to discuss the understanding of their role in mammals and fish.

### 1.1. Estrogen

Estrogen is one of the essential ovarian hormones; it is a pleiotropic hormone and plays an important role in several organs such as the heart, the uterus, and the bone [11–16]. According to the literature, estrogen controls numerous phases of female reproduction, for example, estrus behavior, the increase in serum gonadotropin concentrations, ovulation, uterine propagation, and endometrial gland secretion [10, 14, 17]. Yet again, estrogen impedes osteopenia and is therefore beneficial in the postmenopausal female disposed to osteoporosis. Estrogen essentially binds to its cognate receptor to exert its effects, and in animals, two categories of estrogen receptors (ERs), alpha and beta, exist [4, 5]. A third ER, ER*γ*, has been discovered for teleost fish [18].

### 1.2. Estrogen Receptors

Estrogen receptors are established in several reproductive target tissues, which include uterus and mammary glands of animals and oviduct and liver of oviparous species. Nevertheless, estrogen receptors may also be present in tissues outside the reproductive system [19, 20]. Estrogen receptors are members of the nuclear receptor superfamily of ligand-regulated transcription factors [21]. The life assets of the receptor are originated by binding to the ligand 17*β*-estradiol. An examination of the receptor's mechanism of exploit began ultimately after it was first cloned [18, 22, 23]. In the beginning, it was assumed that there was only one form of the estrogen receptor, but a second form from a distinct gene was momentarily cloned and named estrogen receptor *β* (ER*β*) [20, 24]. The estrogen receptor *α* (ER*α*) was the first estrogen receptor that was characterized and used as an indicator for diagnosis and handling of breast cancer [1, 18, 20, 25]. The estrogen receptor *β* which is generally referred to as ER*β* was discovered in the middle of the 1990s [23, 26]. Estrogen receptors are expressed in several tissues that include certain sections of the brain, bone, adrenal, mammalian gland, epididymis, and thyroid [27, 28]. The two categories of receptors share a high degree of sequence similarity, and the ligand-binding domains have comparable three-dimensional structures [28, 29]. Together, forms of the receptors are highly distributed through several tissues in the body.

The ER*α* is mostly expressed in the uterus, liver, kidney, and heart, while ER*β* is mainly in the ovaries, lung, gastrointestinal tract, bladder, and hematopoietic and essential nervous system [20, 30]. Estrogen signaling plays a vital role in several processes of physiology that include regulation of improvement, development, and function in numerous organ classifications in the body [31]. In fish, three models have been identified: ER*α*, ER*β*, and ER*γ*. The ER*γ* form in fish appears to be closely related to ER*β* which suggests that a gene replication event has transpired within the teleosts. As a result, ER*β* and ER*γ* have been named ER*β*1 and ER*β*2 [1, 4, 5].

### 1.3. Structure of Estrogen Receptors

ER*α* and ER*β* encrypting genes are situated on several chromosomes (6 and 14, respectively) [26, 29, 32, 33], having a distinctive NR domain organization and sharing relatively high protein domain homology. They have “N-terminal of the A/B domain which has a transactivation function and the C domain which contains two zinc finger motifs formed by some cysteine residues that are necessary for DNA binding.” They also have an area which is the hinge region and enables the protein to change its conformation called D area. They have a domain which is possibly the ligand-binding domain and and the F domain. The function and contribution of the F domain to estrogen receptor (ER) action are still not completely understood and sometimes even being referred to as the E domain [34–36]. The A and B protein domains encompass the spots for phosphorylation and ligand-independent transcription stimulation function-1 (AF-1). The C domain comprehends the DNA-binding domain (DBD). The D domain comprises nuclear localization sequences. The E domain is the ligand-binding domain (LBD), and it contains the ligand-dependent transcription activation function-2 (AF-2) [37–40]. The F domain is involved in coregulator recruitment. According to [11, 41], ER*α* and ER*β* share 96 percent amino acid uniqueness in the DBD, nearly 53 percent “amino acid identity in the LBD and 30 percent or less in other domains, involved in transactivation and localization. Also as discussed by [20, 28], it is anticipated that in cell types where two receptor subtypes are coexpressed, the establishment of *α*/*β* heterodimers plays a significant role in estrogen signaling, affecting patterns of gene directive divergent from those controlled by the ER homodimers.” Together, ER full-length mRNAs are encoded by eight exons and are expressed in a diversity of isoforms, owing to alternative splicing [35, 38]. “Estrogen- (E-) bound ER binds to DNA sequences in the promoter regions of target genes at estrogen response elements (ERE) and works as a transcription factor in the nucleus.”

### 1.4. Membrane Receptors Specific to Estrogens

There are several indications which show that both ER*α* and ER*β* are located in the cell membrane [42, 43]. These two membrane-related receptors may occur either in the form of heterodimers (ER*α*-ER*β*) or homodimers (ER*α*-ER*α* or ER*β*-ER*β*) [34, 43, 44]. The present understanding shows that the nuclear estrogen receptor and membrane estrogen receptor do not differ because electrophoretic mobility, spectra, weight, and affinity to E2 are virtually identical in nER and mER [45]. Additionally, examinations with monoclonal antibodies show the relationship among the protein epitopes in two receptor types [43, 44]. Nevertheless, numerous factors can affect the immune detection of mERs. Latest studies on the mER*α* identified a number of undesirable controllers of its expressions such as too many cell passages or too high cell density, lack of cell cycle synchronization within cell population, and serum starvation [46, 47]. Also, integrity of the cell membrane, interactions with other molecules, and flexibility of the epitopes should also be taken into consideration when developing immune-labeling protocols [46, 47].

In addition to “mER*α* and mER*β*, a small number of other proteins were categorized as mER within the past two decades. Among them, the most studied is the G-protein-coupled receptor 30, a rhodopsin-like protein unconnected to steroid nuclear receptors.” In recent times, it has been reported that G-protein-coupled receptor 30 is not only involved in speedy controlling mechanisms, for example, the mobilization of kinase or calcium activation but also play a substantial role in transcriptional activation of genes such as c-fos [44, 48]. A different receptor, functionally distinct form of ER*α* and ER*β*, was identified by Almey et al. [49] and was named ER-X. ER-X can be illustrious from previously labeled receptors inter alia by its molecular weight which is 63 kDa, while the molecular weight of ER*α*, ER*β*, and Gpr30 was 67 kDa, 60 kDa, and 44 kDa, respectively [50]. Additionally, it does not involve in the stimulation of extracellular signal-controlled kinases in the presence of selective estrogen receptor agonists and responds more powerfully to 17*α* than 17*β* estradiol [45]. There was an another discovery of receptor in T47D and MCF-7 cells by Kampa et al. [50] which are related to cell signaling, apoptosis, and transcriptional regulation [51]. The receptor was surprisingly named ERx even though there is no proof that ER-X and Erx are associated. Nonetheless, both receptors are yet to be comprehensively examined [43].

Although the details of the relationship of estrogen receptors with the membrane remain “unclear, two core interactions are understood to be critical: the palmitoylation of these ERs and their interaction with membrane/cytoplasmic scaffolding proteins.” [52] “Palmitoylation is the process of posttranslational lipid modification which increases receptor hydrophobicity and, therefore, enables its anchoring in the membrane. The second interaction apparently occurs between receptors and certain proteins, especially caveolin—integral membrane proteins placed within the area of the plasma membrane invaginations called caveolae. Caveolae are specialized lipid-ordered domains which comprise numerous signaling molecules, especially kinases. Although most studies concern only mER*α* and mER*β*, the association between caveolin-rich light membranes complexes and mERs was also emphasized for ER-X as a way of rapid interaction with the mitogen-activated protein kinase (MAPK) cascade and other signaling pathways.” [49] The method of mER operation is not fully clear yet. Nonetheless, it is recognized that dimerization and internalization play an important role in controlling this development. Since activation follows the dimerization of nERs, it is not astonishing that a similar procedure occurs in their membrane-related equivalents [45, 52, 53]. Mostly, mERs are presumed to be a multiprotein complex that necessitates the collaboration with several molecules including epidermal growth factor receptor (EGFR), insulin-like growth factor 1 receptor (IGFR), and proteins involved in the MAPK pathway such as Ras protein and adaptor protein Shc [45]. Li et al. discovered the exact mechanisms of mER*α* action in human endothelial cells [52, 53]. According to them, palmitoylated mER dimers activate endothelial nitric oxide synthase (eNOS) after E2 stimulation, in cooperation with heat-shock protein Hsp90, Akt kinase, c-Src, and PI-3 K, which results in additional production of nitric oxide [53]. “Alternatively, E2 may induce the detachment of G-proteins into two subunits: G*α* and G*β*.” [45] Worth mentioning, the existing mechanism is about mER*α* only. Therefore, comprehensive research of the mechanisms of estrogen exploit necessitates more broad research in view of the exact membrane and nuclear estrogen receptors and the nonspecific interactions [50].

### 1.5. Estrogen Receptor Binding Affinities and Activation

Estrogen receptors have been identified conventionally as mammal-specific. The preliminary classification of estrogen receptors in mammals showed, fairly astonishingly, that even though mollusks have noticeable estradiol heights, their estrogen receptors could not affect estradiol, nor triggered by it. As an alternative, they exhibit constitutive action [13, 54]. Nonetheless, estrogen receptors lately categorized in double annelids do antagonize and are triggered by estradiol [10, 13, 55, 56]. It seems estrogen receptor constitutive action establishes in mollusks advanced within that heredity. On the basis of the mammal lineage, an amphioxus family estrogen receptor does not bind estrogen receptor estradiol nor it is activated by estradiol [10, 18, 56, 57]. Interestingly, a speculative receptor estimated to the hereditary receptor for androgens, progestin, and corticosteroids activated via estrogens in which initiation stands competitive inhibition by expected hereditary estrogen receptor [58, 59]. Additionally, perhaps the nuclear receptors advanced out of the ligand-stimulated hereditary receptor by means of fatty acids as possible inherited ligands [19, 24, 25, 27, 29, 56]. In complex vertebrate genetics, a noticeable characteristic of estrogen receptors is the ability of them to disturb estrogens by greater affinity. Human estrogen receptor-alpha fixes radio-branded estradiol at roughly quartile time's superior affinity than estrogen receptor-beta. In dissimilarity, in *Micropogonias undulates*, the estrogen receptor-beta types have greater affinity which aimed at estradiol as compared to estrogen receptor-alpha [13, 25, 56, 60]. The *Homo sapien* estrogen receptor-alpha exhibited no variation in affinity [30].

The features of teleost species, estrogen receptor-beta types being capable of binding estradiol with greater affinity than estrogen receptor-alpha have now been defined in other fish types, explicitly, the *Danio rerio* and *Ictalurus punctatus* [13, 26, 28]. Nevertheless, teleost estrogen receptors seem to bind estradiol per fewer affinity than animal species. The affinity of *Oncorhynchus mykiss* estrogen receptor-alpha for estradiol is larger at 4°C than at 22°C [30]. The popular technique of finding estrogen receptor action is the utilization of the receptor-reporter system, in which an activated estrogen receptor will bind an estrogen receptor estradiol and a transactivate reporter gene is measured. One foremost apprehension concerning receptor-reporter evaluates, they characteristically worked in animal cell lines [61]. The cells then possess mammalian co-regulators with fish estrogen receptors. Inside animals, it is currently well recognized that selective estrogen receptor modulators (SERMs) can behave as whichever initiates a physiological response or adversaries to the estrogen receptor, reliant on cellular perspective [33]. There are beliefs that tissue-specific co-regulator manifestation or abundance oversees these variances [33, 62]. About the abovementioned, varying one cofactor is enough to completely reinverse actions of tamoxifen [63, 64]. Consequently, the cell line utilized for the assays is a significant deliberation in deducing the resulting statistics. There are considerable exertions on estrogen receptor utility in fish, especially in rainbow trout estrogen receptor-alpha. Using a receptor-reporter method with yeast cells, established that *Oncorhynchus mykiss* estrogen receptor-alpha has greater base action linked to the human estrogen receptor. According to [30], the concentrated action of *Oncorhynchus mykiss* estrogen receptor alpha on a reporter per estrogen receptor estradiol only extended twenty-six percent of the human estrogen receptor. Nonetheless, on a reporter per three estrogen receptor estradiol *Oncorhynchus mykiss* ER*α* and human ER-alpha demonstrated comparable action. The focus on variances in estrogen receptors among vertebrates that may be flouted by depending exclusively on a single receptor reporter evaluate. Through generating fantasies among the *Oncorhynchus mykiss* ER*α* and human estrogen alpha, it is consequently established amino acids in the DNA-binding domain are accountable for a pronounced opinion that *Oncorhynchus mykiss* estrogen receptor alpha. This might only extend an extreme activating 74 percent lesser than human estrogen receptor alpha on reporter hypothesis per one estrogen receptor estradiol [63, 65]. Owing to the well-preserved state of the BDB section, the researchers settle that the variance is probably owing to 7 amino acid variances and extra Arg in *Homo sapien* estrogen receptor alpha spot 260. In the argument of the researchers, they define initial finding that incompletely incriminates the supplementary Arg in *Homo sapien* matched to fish estrogen receptor alpha. Furthermore, the different effects perceived among reporters of 1 estrogen receptor estradiol against three estrogen receptor estradiol indicates that the *Oncorhynchus mykiss* estrogen receptor needs the steadiness of DNA through protein-protein communications, of which these communications might be facilitated by the AF-1 domain [66]. Inside the AF-1 domain of the *Oncorhynchus mykiss* estrogen receptor, negligible section eleven amino acids situated from the beginning of B domain which is essential for AF-1 action [67]. The designs of the eleven amino acids displayed the establishment of alpha-helix which is well-preserved through mammals. Interestingly, the researchers established that A domain of *Oncorhynchus mykiss* estrogen receptor alpha plays a hindering role, probably through the collaboration of the C-terminal section of the estrogen receptor [67]. A multitude of co-controllers has been established to interrelate per and modify the estrogen receptors in mammals [62]. There is no doubt that considerable extra effort is vital to distinguish the teleost coregulators complicated in estrogen receptor signaling appropriately. It will afford significant background to define receptor-reporter assays. Comparable to *Homo sapien* estrogen receptor alpha, the *Oncorhynchus mykiss* estrogen receptor alpha is triggered, in yeast functional unit, through choosy ER modulator, 4-hydroxytamoxifen, but seemingly not through pure antiestrogen, ICI 164 384, even though estrogen receptor action did seem to upsurge with dose of ICI 164 384 [13, 30]. It is also significant in the direction that other endocrine aspects, like the androgens and thyroid hormones, also have been demonstrated to control the distribution of the estrogen receptors [61].

### 1.6. Significance of Estrogens and Estrogen Receptors

#### 1.6.1. Metabolic Effects

Estrogens are important in maintaining bone mass primarily by retarding bone resorption. They control the action of parathormone, a hormone which intakes calcium ions from bones and teeth, thus maintaining the calcium balance. They also cause salt and water retention that is edema. Additionally, blood coagulation is increased due to the formation of various clotting factors and also it promotes vasodilation by inducing secretion of nitric oxide synthase (NO) and PGI2. It also increases plasma “sex hormone binding globulin (SHBG), thyroxine binding globulin (TBG), and cortisol binding globulin (CBG).” [68, 69]

#### 1.6.2. Developmental Actions in Humans

Estrogens are accountable for the advancement of pubertal changes and secondary sexual characteristics. They cause the growth and development of uterus, vagina, and fallopian tubes as well as also contribute to breast enlargement in females (humans). In addition, they add to the molding of the body contours and are responsible for the pubertal development spurt of the long bones and epiphyseal closure [70].

Undoubtedly, estrogens play key roles in female hormone regulation and signaling and are responsible for metabolic, behavioral, and morphologic changes occurring during the stages of reproduction. They are also involved in growth, development, and homeostasis of a number of tissues. Again they also regulate reproduction, transport, and concentration of testicular liquid and anabolic activities of androgens in males [71, 72].

Estrogens have beneficial effects in preventing heart diseases, osteoporosis, and Alzheimer's disease; however, there is also evidence that estrogens may promote cancer of the breasts, uterus, and other organs. Estrogen may cause adverse effects such as risk of thromboembolic events, postmenopausal uterine bleeding, and nausea, and breast tenderness [73].

#### 1.6.3. The Activities of Estrogen and Estrogen Receptors in Humans and Fish

In vertebrates, the activities of estrogens are applied through ERs (estrogen receptor alpha and estrogen receptor beta), and have shown that their expression profiles are tissues and cells specific. In mammalian immune cells, estrogen receptors (ERs) are receptive to estrogens. It must be noted that in recent times, ERs are also found in the teleost fish cells and immune organs, indicating estrogen immunomodulatory function conservation all through the evolution of vertebrates. In trout immune system, low mRNA numbers of ERs were reported with limited estrogen responsiveness. This is an indication that the estrogen activities on trout immunity might not be principally facilitated through genomic activities, though it may comprise other mechanisms [74].

The principal physiological estrogen function in vertebrates is regulation of sexual growth and propagation. Nonetheless, estrogens possess pleiotropic utilities away from the conventional utility in the propagative bloc, they influence many other systems of physiology which include the immune system [74, 75]. The estrogens' immunomodulatory activities in animals differ with reverence to organism's condition of physiology, cell type or estrogen concentrations [76–78]. Specifically, the status of female reproductive and related variations of “estrogen and the levels of its estrogen receptor possesses key influence on the immune system response to estrogens.” [79]

Within the immune system of trout fish, estrogen receptor alpha was found to express conspicuously, whilst ER*β*2 was also found expressing with the same levels of ER*α* in the organs such as spleen and head kidney indicating a significant starring role of these ERs in teleost immune systems [74].

Many studies reported on ERs in immune system using quantification of relative mRNA method for several teleost fishes such as seabream (*Sparus aurata*), which was reported to have no copies in the head kidney acidophilic granulocytes and testicular, however, “head kidney contained ER*α* mRNA, but absence of ER*β*1 or ER*β*2 mRNA, in channel catfish (Ictalurus punctatus),” [80] ER*α* and ER*β* mRNA was found in head kidney and spleen, whereas only ER*α* was present in “peripheral blood leukocytes, in carp (Cyprinus carpio),” [80] ER*α* mRNA was highly expressed while there was no ER*β* presence in peripheral blood leukocytes there was also a report of low-level ER*β* in isolated leukocytes in head kidney. Accordingly, the tissue leukocytes exhibited high expression levels of ER*α* than of ER*β* in all species mentioned above and were totally lacking in peripheral blood leukocytes [81–83]. In contradiction to the mentioned species above, [74] reported the presence of both ER*β* forms in the rainbow trout's peripheral blood leukocytes and made some interesting revelation that the ER*β*2 mRNA figures were the same as those of ER*α* in the head kidney and spleen. Therefore, we can conclude from numerous studies on nuclear ER that estrogen receptor alpha is the main estrogen receptor in the immune system of teleost of diverse species, “but not in the immune organs essentially while estrogen receptor beta expressions in the fish immune system seems to differ with the origin of the cells and across species.” [74]

It must be noted that in mammals, estrogens such as 17*β*-estradiol (E2) modulates the functioning of immune cells, activation, differentiation, development, and life span, and can have both immunosuppressive and immunostimulating actions [76, 84, 85]. Mostly, ER*α* form has a more noticeable expression and distribution in the immune system of mammal's immune cells than estrogen receptor beta [85].

Prostate tissue manifested both estrogen receptor beta and estrogen receptor alpha and [86–88]. Immunohistochemical researchers have discovered that specimens of prostate cancer manifested both estrogen receptor beta and estrogen receptor alpha, even though at different levels different phases of prostate cancer still remain mysterious [89].

Several studies on a role “of estrogen receptor alpha or estrogen receptor beta in prostate cancer have been” carried out and linger on. Many of these discoveries recommend that ER*α* behaves as an oncogene which facilitates the harmful influences of estrogen, such as prostate carcinogenesis, inflammation, and proliferation [90]. In dissimilarity, ER*β*, which manifestation declines by promoter DNA methylation as prostate cancer advancements, appears to play “anti-oncogenic role.” Certainly, estrogen receptor beta agonist has been reported to have averted the propagation of prostatic epithelium [91]. Once more, the opposite role of estrogen receptors in the prostate tissues revolution was proven by Ricke and colleagues by means of estrogen receptor alpha or estrogen receptor beta knockout mice cured with combinations of androgen and estrogen. Whereas “ER*β* knockout prompted dysplastic variations and premalignant revolution, ER*α* knockout mice persisted prostate cancer-free.” [90] Some discoveries, nonetheless, are backing the defensive starring role played by ER*β* in a prostatic revolution. For example, the loss of estrogen beta manifestation shows a relationship, with amplified propagation in the “ER*β* KO/transgenic adenocarcinoma of mouse prostate classic” [92] and human prostate cancer specimens [80, 93, 94].

#### 1.6.4. The Role of Estrogen Receptors in the Teleost Brain

Steroid hormone receptors are present in vital areas of brain identified to control social behavior in other vertebrates including “the mammalian amygdalar complex, hippocampus, striatum, preoptic area, anterior hypothalamus, ventromedial hypothalamus, and ventral tegmental area.” [95] For example, in *A. burtoni* ER*α* and ER*β* extensively expressed all through the telencephalon and diencephalon though the ER*β*2 subtype seems to be distributed more than ER*β*1, which are more than earlier available or reported in several teleost species, even though several of those literature studies define mRNA but not protein [95]. The mRNA ER*α* expressions or distributions have been studied in the midshipman [96], Atlantic croaker [97], rainbow trout [98], zebrafish [26], and eelpout [99], while that of ER*β*1 and ER*β*2 mRNA subtypes has been studied in the Atlantic croaker [97], sea bass [100], and zebrafish [26].

Several lesion and stimulus studies have point to the preoptic area (POA) as area of brain that controls antagonistic and reproductive activities in teleosts, in agreement with other studies in other vertebrates [101, 102]. According to [103], the electrical stimulation of the preoptic area proliferate wooing behavior and reduces antagonistic exhibition in Lepomis sunfish males. For a male fish, it is beneficial to it to reduce its hostile exhibitions in wooing a female counterpart [104]. In male killifish, *Fundulus heteroclitus* ablation of the preoptic area reduces the spawning reflex [105]. According to [95], all sex steroid hormone receptors, that is, ER*α*, ER*β*1, and ER*β*2, were present inside the three cell groups that are parvocellular, magnocellular, and gigantocellular composed of the preoptic area. These three cell sets could differentially control “social dominance, as dominant males have higher AVT expression in the gigantocellular subregion compared to subordinate males” according to [106]. Similarly functionally vital by means of a social signal to several male teleost fish such as *A. burtoni* and Lepomis sunfish is the generation of body pigmentation [95, 107], and greater heights of flowing testosterone [106, 108]. There is a need for further studies on estrogen receptors in the diverse subpopulations of the preoptic area to elucidate whether these receptors are in a different way controlling social dominance in an area-specific modus by using quantitative discovery techniques and pharmacological controls to further elucidate the starring role of estrogen receptors in controlling the outstanding plasticity of social behavior of teleost species.

## 2. Conclusion and Future Directions

Vertebrates and other mammals including species of teleosts have no doubt become an appropriate and widely held suitable species to study the activities and function of estrogens and estrogen receptors. Estrogen receptor subforms expressed broadly, and hepaticvitellogeneisis offers a physiologically significant endpoint of estrogen receptor activation. Certainly, hepatic vitellogenesis and estrogen receptor alpha gene's distribution are frequently utilized as bioassays aimed at endocrine disruptors that have estrogen-like action [10, 61]. It is even more critically important that physiological background must cautiously be well-thought-out when scheming such experiments and assessing ERs data. Importantly, as established in mammals as well as cancer cell lines, there is an essential need for biologists to continue to ascertain estrogen receptor interrelating co-factors, their actual functions, tissue distribution, and their regulation as well as dimerization between the estrogen receptors. In addition to the impact on the establishment of secondary sexual features, estrogens also have an influence on several cell procedures. Estrogens control apoptosis, migration, growth, and proliferation of cells [95]. Nonetheless, the current signal indicates that the conventional genomic and the nongenomic technique of estrogen could be assimilated by the swift collaboration of estrogen receptors with specific binding partners. It must also be noted that nongenomic effects of estrogens are multifaceted and have not yet completely elucidated.

There is also a need to investigate whether the exposure to exogenous estrogens from various sources could trigger cancer development in experimental animals including fish. This has become necessary due to studies suggesting that the cancer-causing effect of estrogens and metabolites of some estrogens is linked with the stimulation of estrogen receptors alpha and beta that activate transcription of genetic material stimulating cell propagation. Additionally, numerous cancers such as ovarian cancer, uterine cancer, and breast cancer which are mostly associated with a female are mostly suggested to be causative of estrogens. We believe that the increased understanding of the molecular signaling nature of ERs will also flourish our capacity to precisely target the receptor and its signaling pathways for varied arrays of research.
